# Supplementary material for: Genetic Factors Involved in Cardiomyopathies and in Cancer
Source: J Clin Med. 2020 Jun 2;9(6):1702. doi: 10.3390/jcm9061702 (PMC7356401; doi:10.3390/jcm9061702)
Supplement: Supplementary file 1 [file jcm-09-01702-s001.pdf]

Table 1 Supplementary. Interactions obtained by crossing cardiac genes with the KEGG terms related to oncological molecular pathways.

ID	KEGG term	Genes
hsa04918	Thyroid hormone synthesis	TTR
hsa04630	JAK-STAT signaling pathway	FHL1
hsa04630	JAK-STAT signaling pathway	PTPN11
hsa04010	MAPK signaling pathway	FLNC
hsa04510	Focal adhesion	FLNC
hsa04510	Focal adhesion	MYL2
hsa05205	Proteoglycans in cancer	FLNC
hsa05205	Proteoglycans in cancer	PTPN11
hsa01100	Metabolic pathways	GLA
hsa04142	Lysosome	GLA
hsa04142	Lysosome	LAMP2
hsa05200	Pathways in cancer	JUP
hsa04140	Autophagy – animal	LAMP2
hsa04145	Phagosome	LAMP2
hsa04210	Apoptosis	LMNA
hsa04022	cGMP-PKG signaling pathway	MYH7
hsa04022	cGMP-PKG signaling pathway	PLN
hsa04530	Tight junction	MYL2
hsa04530	Tight junction	PRKAG2
hsa04670	Leukocyte transendothelial migration	MYL2
hsa04670	Leukocyte transendothelial migration	PTPN11
hsa04024	cAMP signaling pathway	PLN
hsa04024	cAMP signaling pathway	TNNI3
hsa05206	MicroRNAs in cancer	TPM1
hsa04068	FoxO signaling pathway	PRKAG2
hsa04152	AMPK signaling pathway	PRKAG2
hsa04211	Longevity regulating pathway	PRKAG2
hsa04650	Natural killer cell mediated cytotoxicity	PTPN11
hsa05211	Renal cell carcinoma	PTPN11
hsa05220	Chronic myeloid leukemia	PTPN11
